# The transcription elongation factors Spt4 and Spt5 control neural progenitor proliferation and are implicated in neuronal remodeling during *Drosophila* mushroom body development

**DOI:** 10.3389/fcell.2024.1434168

**Published:** 2024-10-09

**Authors:** Lea Barthel, Stefani Pettemeridi, Ali Nebras, Hayley Schnaidt, Karoline Fahland, Lea Vormwald, Thomas Raabe

**Affiliations:** Department Molecular Genetics of the Faculty of Medicine, Biocenter, University of Würzburg, Würzburg, Germany

**Keywords:** *Drosophila*, DSIF complex, mushroom bodies, neuroblast, neuronal remodeling

## Abstract

Spt4 and Spt5 form the DRB sensitivity inducing factor (DSIF) complex that regulates transcription elongation at multiple steps including promotor-proximal pausing, processivity and termination. Although this implicated a general role in transcription, several studies pointed to smaller sets of target genes and indicated a more specific requirement in certain cellular contexts. To unravel common or distinct functions of Spt4 and Spt5 *in vivo*, we generated knock-out alleles for both genes in *Drosophila melanogaster*. Using the development of the mushroom bodies as a model, we provided evidence for two common functions of Spt4 and Spt5 during mushroom body development, namely control of cell proliferation of neural progenitor cells and remodeling of axonal projections of certain mushroom body neurons. This latter function is not due to a general requirement of Spt4 and Spt5 for axon pathfinding of mushroom body neurons, but due to distinct effects on the expression of genes controlling remodeling.

## Introduction

Transcription by RNA Polymerase II (Pol II) is a multi-step process that involves the assembly of distinct protein complexes to control transcription initiation, pausing, elongation, and termination. One of these transcription-regulating factors is Spt5. It was initially identified in *Saccharomyces cerevisiae* in a genetic screen as a mutation that suppresses the phenotype of a Ty insertion mutation in the 5′ noncoding region of the HIS4 gene (Winston et al., 1984). Spt5 proteins (NusG in bacteria) are found in all three domains of life and share a single N-terminal NusG (NGN) and at least one KOW domain, with eukaryotic Spt5 proteins containing up to seven KOW domains (KOW1-7), a N-terminal acidic region and a C-terminal repeat region (CTR). Except for bacteria, Spt5 dimerizes through its NGN domain with the zinc-finger protein Spt4 to form a complex called DRB sensitivity-inducing factor (DSIF). DSIF fulfills multiple functions during transcription. It stabilizes Pol II and promotes promotor-proximal pausing of Pol II. Pol II together with Spt4 and the Spt5 NGN and KOW1 domains form the DNA exit tunnel. The RNA exit site is formed by Pol II and Spt5 KOW4 and 5 domains. The paused state is stabilized by Negative Elongation Factor (NELF), which contacts Spt4 and the Spt5 NGN domain at the DNA exit tunnel and also extends to Spt5 KOW 1–4 (Decker, 2021; Dollinger and Gilmour, 2021; Song and Chen, 2022). The structural and biochemical data were complemented by structure-function analyses in *Drosophila* showing that transgenes with deletion or mutations in KOW1, 4, 5 or the NGN domain failed to complement loss of endogenous Spt5 function (Qiu and Gilmour, 2017; Dollinger et al., 2023). One major regulatory mechanism for release of paused Pol II and transcription elongation is phosphorylation of NELF, Pol II and Spt5, which results in phase transition of Spt5 and Pol II from pausing clusters into liquid-like elongation droplets (Guo et al., 2023). This is accompanied by conformational changes of the KOW domains contacting DNA and RNA. Spt4 and Spt5 interact with elongation factors to increase processivity of transcription and promote elongation through nucleosomes. Through less clarified mechanisms, Spt5/DSIF are also involved in Pol II transcription termination. In addition to their function in Pol II transcription, Spt4 and Spt5 interact with RNA polymerase I for ribosomal RNA synthesis (Decker, 2021; Song and Chen, 2022).

Whereas the molecular properties of DISF are well studied, its role in the context of cell type specific functions during development or disease processes in multicellular organisms is less clear. Although mainly considered as a general transcriptional regulator, there are examples of more gene specific functions. Decreasing expression of Spt4 or its interaction with Spt5 selectively lowers the transcription of genes with long tri-or hexanucleotide repeats associated with Huntington disease (Htt, (Liu et al., 2012; Cheng et al., 2015; Deng et al., 2022)), amyotrophic lateral sclerosis/frontotemporal dementia (C9orf72, (Kramer et al., 2016)) and spinocerebellar atrophy type 36 (Nop56, (Furuta et al., 2019)).

Mimicking the effects of Spt5 knock-down with small-molecule inhibitors identified target genes that responded with diverse kinetics and uncovered Spt5 regulatory roles in metabolism and processing of histone genes (Bahat et al., 2019). In zebrafish, complete loss of Spt5 had far-reaching effects on embryonic development (Keegan et al., 2002). Comparative expression profiling of wild-type and Spt5 mutant zebrafish embryos showed that only 5% of genes are differentially expressed (Krishnan et al., 2008). A single amino acid substitution in zebrafish Spt5 resulted in reduction in the number of dopaminergic neurons and a corresponding surplus of serotoninergic neurons (Guo et al., 2000).

In *Drosophila*, Spt5 is located at sites of active transcription on polytene chromosomes and becomes recruited to heat-shock induced genes (Andrulis et al., 2000; Kaplan et al., 2000), but also controls transcriptional activation and repression of specific genes during embryogenesis (Jennings et al., 2004). Spt5 is also required for gene dosage compensation in male flies by promoting X-chromosomal gene expression (Prabhakaran and Kelley, 2012). In a *Drosophila* brain tumor model, knock-down of Spt5 impaired proliferation of tumor inducing neural progenitor cells, delayed tumor formation in the adult and increased lifespan (Hofstetter et al., 2024).

Available data indicated the essential requirement of Spt4 and Spt5 for viability, which hampered the phenotypic analysis of complete loss-of-function alleles at late development stages or in the adult. Mutational analysis of both genes in the context of a specific developmental process should provide information about identical or distinct cellular phenotypes. To address these points, we generated Spt4 and Spt5 knock-out flies and analyzed the phenotypic consequences using the well-studied mushroom body (MB) development in the fly brain as a model (Lin, 2023). Briefly, three major classes of MB neurons (Kenyon cells, KCs) are generated from four equipotent neural progenitor cells (mushroom body neuroblasts, MBNB) in one brain hemisphere in a chronological order; γ-KCs are generated from embryonic stages until early third instar larvae, followed by α´/β′-KCs until late third instar larvae and finally α/β-KCs are born until MBNB terminate proliferation around mid-pupal stage (Ito et al., 1997; Lee et al., 1999). KCs are named according to their axonal projections in the adult brain; α´/β′-and α/β-KCs bifurcate their axons to form the vertical α´-/α-lobes and the medial β´-/β-lobes, respectively. While the axonal projections of α´/β′- and α/β-KCs are maintained throughout life, axons from γ-KCs undergo developmental remodeling at the transition from larval to pupal stage. The initial bifurcated axonal branches are pruned and regrow only into medial direction to form the adult-specific γ-lobe. De-and regeneration of γ-KC axons has been studied in detail and depends on cell intrinsic and extrinsic mechanisms including glia-neuron interactions and signaling, engulfment of axonal debris by infiltrating glial cells, upregulation of protein degradation pathways, transcriptional changes induced by the steroid hormone 20-hydroxyecdysone (Ecdysone), upregulation of growth promoting pathways and re-organization of the actin cytoskeleton (Yaniv and Schuldiner, 2016; Furusawa and Emoto, 2021; Boulanger and Dura, 2022; Truman and Riddiford, 2023).

In this study we show that Spt4 and Spt5 are required for MBNBs proliferation and we uncover a novel function of both proteins in controlling remodeling of γ -KC axons through differential effects on gene expression.

## Materials and methods

### General fly work

Flies were maintained at 25°C on standard cornmeal food in a 12-h dark-light cycle. The following fly strains were obtained from Bloomington *Drosophila* Stock Center (IN, United States): *ey* ^*OK107*^ *-Gal4* (BL#854), *UAS-mCD8::GFP* (BL#5130 and 5137), *P[w* ^*+*^ *; FRT]G13 (FRT42B)* (Bl#1956), *heat-shock promotor* (*hsP*)*-Flp; FRT 42B, UAS-mCD8::GFP* (Bl#5131), *FRT 42B, tubulin promotor (tubP)-Gal80* (Bl#5140), *UAS-shRNA-Spt5* (Bl#34837), H24-Gal4 (Bl#51632) and worniu (wor)-Gal4 (BL#56553). The *hs-Flp, UAS-mCD8::GFP* fly line was a kind gift from P. Gallant, the combined tubP-Gal80 (ts); GMR-71G10-Gal4 fly stock was generously provided by Oren Schuldiner. For MARCM analysis (Lee et al., 1999), mitotic recombination between *FRT* sites was induced in first instar larvae by a 2-h heat shock (37°C) and animals of the genotype *hsP-Flp, UAS-mCD8::GFP; FRT42B, Spt5* ^*Δ*^ *(or Spt4* ^*Δ*^ *)/FRT42B, tubulin promotor (tubP)-Gal80; ey* ^*OK107*^ *-Gal4* were selected for further analysis. In control animals, *UAS-mCD8::GFP* localizes on the second chromosome. Homozygous MBNB clones were detected by mCD8::GFP expression in third instar larval or adult brains.

### Generation of Spt4 and Spt5 CRISPR alleles

#### gRNA cloning

CRISPR/Cas9 cutting sites within the *Spt4* and *Spt5* transcription units were identified with the *CRISPR Optimal Target Finder*. Complementary 5′-phosphorylated oligonucleotides with target-specific sequences for Spt4 gRNAs (gRNA1: 5′-GGC​CTT​TGA​CGC​GAT​ACC​CA-3´; gRNA2: 5′-TAC​GTG​ACA​TGA​AGA​ATC​GT-3´; sequences are in 5′–3′ order of the transcript) or Spt5 gRNAs (gRNA1: 5′-AAC​GTG​GGT​AAT​CTT​CGG​AT-3´; gRNA2: 5′-GTT​GGT​TAC​ATG​AAC​ACT​CC-3′) were synthesized with matching overhangs for directional cloning into the BbsI cut *pU6-BbsI-chiRNA* vector (Gratz et al., 2015).

### Generation of HDR donor plasmids

As template DNA for homology arm cloning for Spt5 we used the P[acman] BAC clone CH321-94A4 (Venken et al., 2009) obtained from BACPAC Resources Center (Oakland, CA, United States). A 1 kb ´fragment encoding the 3′-homology arm was amplified with forward primer 5′-GTT​ACA​TGA​ACA​CTC​CGT​CG-3′ and reverse primer 5′- AAG​AAG​GAA​AGG​ATA​GTG​TG-3′, both containing overhangs for directional cloning after SapI digestion into the pHD-DsRed-attP vector (Gratz et al., 2015). This construct was digested with AarI and the 5′-homology arm (1 kb) amplified with forward primer 5′ AAT​ATG​TCG​GAT​AGT​GGC​TC-3′ and reverse primer 5′-CGA​AGA​TTA​CCC​ACG​TTA​TC-3′ was inserted after digestion of the flanking AarI linker. For generation of the Spt4 HDR donor plasmid, we followed the same strategy but used genomic DNA isolated from *w* ^*1118*^ flies as template for amplification of the 0.86 kb 5′-homology arm (AarI forward primer: 5′-CTG​AGT​GCA​TAG​CAA​ACG​GAG-3′, AarI reverse primer: 5′-CGC​GTC​AAA​GGC​CAT​ATT​TAC-3′) and the 0.91 kb 3′-homology arm (Sap forward primer: 5′-GAC​ATG​AAG​AAT​CGT​GGA​ATT​GTC-3, Sap reverse primer: 5′-CAG​GTG​CAG​GTA​GAC​AGC​C-3′). The two gRNA constructs and the HDR donor construct for Spt4 or Spt5 were co-injected by Bestgene Inc. (Chino Hills, CA, United States) or FlyORF Injection Service (Zürich, CH) into embryos carrying a *nos-Cas9* or *vas-Cas9* source resulting in *Spt4 ^Δ,^ ^DsRed+^* and *Spt5 ^Δ,^ ^DsRed+^* flies. Correct gene targeting was confirmed by sequencing of PCR fragments amplified from genomic DNA of adult flies using primers which bind outside the homology arm sequences and pHD-DsRed-attP specific primers. For MARCM analysis, the mutant alleles were recombined onto a *FRT42B* chromosome (*FRT42B,* *Spt4^Δ,^ ^DsRed+^* and *FRT42B,* *Spt5^Δ,^ ^DsRed+^*). Finally, the 3xP3-DsRed marker was removed by expressing a germline Cre source to establish *FRT42B,* *Spt4^Δ,^ ^DsRed−^* (named *Spt4^Δ^*) and *FRT42B,* *Spt5^Δ,^ ^DsRed−^* (named *Spt5^Δ^*) fly stocks, again verified by sequencing (see Supplementary Figure S1).

### Expression constructs for S2R cells and transgenesis

For expression of Myc-tagged Spt5 in S2R cells, the coding sequence of Spt5 was amplified by linker PCR from an existing Spt5 plasmid kindly provided by P. Gallant and cloned into a modified pAC5.1 vector 3′ to the 6xMyc-tag sequences. This construct was used for further subcloning by linker PCR into the pUASTattB vector for transgenesis. HA::Spt4 was synthesized by Invitrogen (Thermo Fisher Scientific, Waltham, MA, United States) and inserted into a pcDNA3.1 vector before subcloning by linker PCR into pUASTattB. The E265K substitution in Spt5 and the S69F substitution in Spt4 were introduced with the Q5 Site directed mutagenesis kit (NEB, Ipswich, MA, United States) using the NEBaseChanger tool for mutagenesis primer design. Transgenic flies were generated by PhiC31 -mediated integration into the third chromosomal attP landing site of fly strain ZH-86Fb loxP (FlyORF Injection Service, Zürich, CH).

### Immunohistochemistry

For immunostainings, brains from late third instar larvae or adults were dissected in PBS (10 mM Na_2_HPO_4_, 2 mM KH_2_PO_4_, 2.7 mM KCl, 137 mM NaCl) and fixed on ice for 25 min in PLP solution (4% paraformaldehyde, 10 mM NaIO_4_, 75 mM lysine, 30 mM sodium phosphate buffer, pH 6.8). After blocking in PBT (PBS plus 0.3% Triton X-100) containing 5% normal donkey serum for 1 h, brains were incubated overnight at 4°C with combinations of the following primary antibodies: rat anti-Chinmo (1:500, N. Sokol, Bloomington, IN, United States), rabbit anti-cleaved Dcp-1 (1:100, # 9578, Cell Signaling Techn., Danvers, MA, United States), mouse anti-Dachshund (1:15, clone mABdac2-3, Developmental Studies Hybridoma Bank (DSHB), Iowa City, IA, United States), mouse anti-EcR B1 (1:25, clone AD4.4, DSHB), mouse anti-Fasciclin II (1:10, clone 1D4, DSHB), chicken anti-GFP (1:1,500; #ab13970, abcam, Cambridge, United Kingdom), rabbit-anti-HA-tag (1: 800, #3724, Cell Signaling Techn.), rabbit anti-Imp (1:1,500, F. Besse, Nice, FR), rabbit anti-Mef2 (1:750, H. T. Nguyen, Erlangen, DE), mouse anti-Myc-tag (1:100, sc-40, Santa Cruz Biotechn., Dallas, TX, United States), rabbit anti-Retinal homeobox (1:750, U. Walldorf, Homburg, DE), guinea pig anti-Sox14 (1:30, S. Rumpf, Münster, DE). Samples were washed 4 times for 1 h in PBT followed by overnight incubation with secondary antibodies conjugated with AlexaFluor 488, Cy3 or Cy5 (Dianova, Hamburg, DE), and at least 4 washes in PBT for 1 h before embedding in Vectashield (Vector Laboratories, Newark, CA, United States). Confocal images were collected with a Leica SPE or SP8 microscope (Leica Microsystems, Wetzlar, DE). Image processing was carried out with the ImageJ distribution Fiji (Schindelin et al., 2012).

For 5-ethynyl-2′-deoxyuridine (EdU) labeling, brains from third instar larvae were dissected in PBS and incubated with 20 µM EdU in PBS for 90 min. Fixation in 4% paraformaldehyde for 15 min was followed by immunostaining as described before, before EdU incorporation into replicating DNA was detected with the Click-iT^®^ Alexa Fluor 647 EdU imaging kit (Invitrogen, Thermo Fisher Scientific).

### Quantification and statistics

For quantification of antibody stainings, signal intensities of 10 randomly selected KCs within GFP labelled control, *Spt4* ^*Δ*^ or *Spt5* ^*Δ*^ MBNB clones in one brain were measured with an ImageJ macro (kindly provided by Nils Reinhardt) and averaged. The same was done with 10 non-clonal KCs in close proximity in the same focal planes and then the signal intensity ratio was calculated. Cells from six individual brains per genotype were measured. Distributions of data did not deviate significantly from normality (Kolmogorov-Smirnov test; *P* > 0.2). A one-way analysis of variance (ANOVA) was performed for statistical analysis. The ratio between clonal/non-clonal cells was considered as dependent variables, and the strain (control versus mutant lines) was considered as an independent variable. For multiple testing within one data set, the level of significance was adjusted with the Bonferroni correction factor. Graphs are presented as Box Plots generated with GraphPad Prism 6. Asterisks depict the level of statistical significance ^****^ *p* ≤ 0.00001.

### Proximity ligation assay (PLA)

Third instar larval brains were fixed and incubated with anti-GFP, anti-HA and anti-Myc antibodies as described before. Incubation with an Alexa488-conjugated antibody for detection of GFP and DNA with Hoechst 33342 (1:2000, Thermo Fisher Scientific) were done for 3 h. The PLA assay was done according to the manufacturer’s operating instructions (Duolink^R^, Sigma-Aldrich, Merck, Burlington, MA, United States) with extended times for the Plus and Minus PLA probes incubation (90 min), ligation (60 min) and amplification (120 min) steps.

### Cell culture, immunoprecipitation and western blot analysis

*Drosophila* S2R cells were maintained at 25°C in Schneider’s *Drosophila* Medium (Gibco, Thermo Fisher Scientific) supplemented with 10% fetal bovine serum (Biochrom, Berlin, DE) and 1% penicillin/streptomycin (Merck, Darmstadt, DE). 2 × 10^6^ cells were seeded on 60 mm dishes and after overnight incubation, transfections with combinations of pAC5.1-Myc::Spt5, pAC5.1-Myc::Spt5^E265K^, UAS-HA::Spt4, UAS-HA::Spt4^S69F^ and tubP-Gal4 plasmids were done with the Effectene Reagent (Qiagen, Hilden, DE) according to manufacturer’s protocol. Cells were collected 24–36 h after transfection in lysis buffer (25 mM Tris pH 7.4, 150 mM NaCl 1 mM EDTA, 1% NP-40, 5% glycerol) and after removal of cell debris by centrifugation (13.000 g for 10 min at 4°C), proteins from the same preparation were either first immunoprecipitated with anti-Myc coupled agarose beads (Pierce, Thermo Fisher Scientific) or directly separated by SDS-PAGE followed by Western blot transfer. Nitrocellulose membranes were incubated overnight at 4°C with rabbit-anti-HA-tag (1: 800, #3724, Cell Signaling Techn.) or mouse anti-Myc-tag (1:1,000, sc-40, Santa Cruz Biotech.) antibodies. Following incubation with HRP-coupled secondary antibodies, signal detection was done with the ECL Plus detection reagents (GE Healthcare Life Science, Buckinghamshire, United Kingdom) and a ChemoCam ECL Imager equipped with a 16bit camera (Intas, Göttingen, DE).

## Results

### *Drosophila* Spt5 binds to and retains Spt4 in the nucleus

**FIGURE 1:**
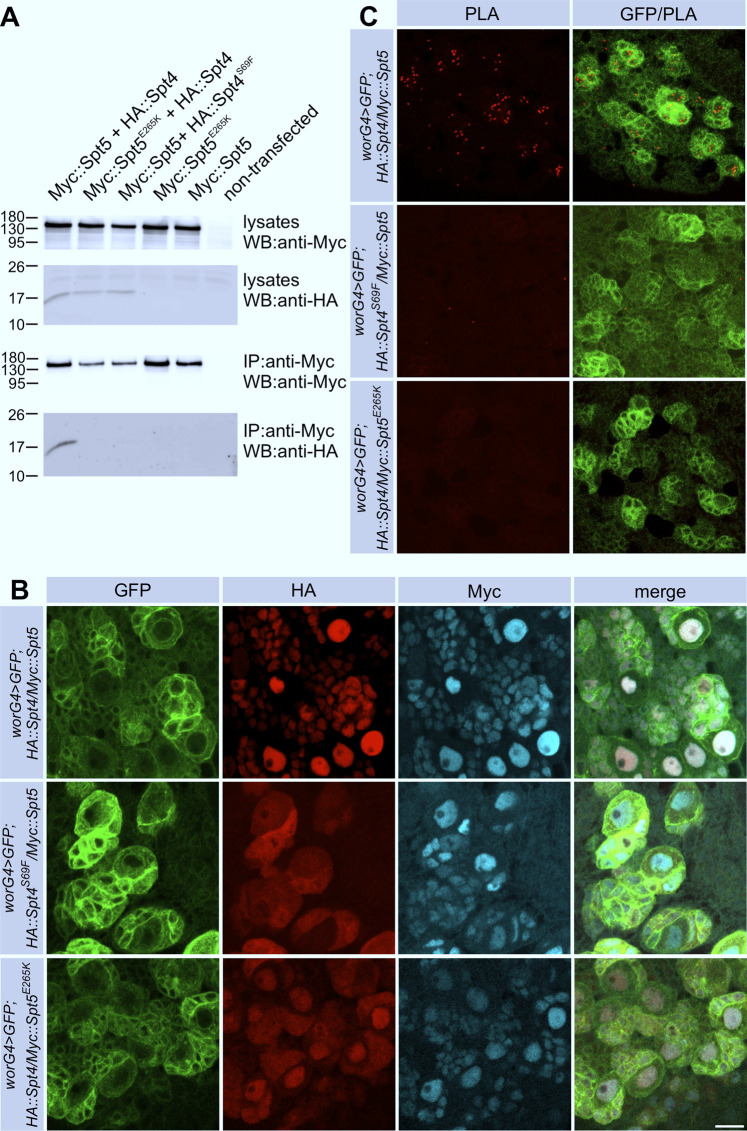
Interaction of Spt4 with Spt5. **(A)** Myc-tagged Spt5 and Spt5^E265K^ were transiently expressed in S2R cells alone or in combination with HA-tagged Spt4 or Spt4^S69F^. Cell lysates were tested for protein expression by Western blot (WB) using anti-HA and anti-Myc-antibodies. For co-immunprecipitation (IP), lysates were first incubated with immobilized anti-Myc antibodies, and after elution, proteins were detected with anti-Myc and anti-HA antibodies. Molecular weight markers (in kD) are indicated. **(B)** Spt4 localization depends on interaction with Spt5. Combinations of UAS-Myc::Spt5, UAS-Myc::Spt5^E265K^, UAS-HA::Spt4 and UAS-HA::Spt4^S69F^ were expressed with worniu-Gal4 (worG4) in neuroblast lineages in larval brains, which in addition were labeled with UAS-mCD8::GFP. Immunohistochemistry was done with anti-Myc (cyan), anti-HA (red) and anti-GFP (green) antibodies. Scale bar: 10 μm. **(C)** Close association of Spt4 and Spt5. PLA signals (red) in neuronal cells indicating interaction are only detected by co-expression of the non-mutated versions of UAS-Myc::Spt5 and UAS-HA::Spt4 with worG4 in combination with UAS-mCD8::GFP (green). Scale bar: 10 μm.

In a previous work, Guo et al. determined the crystal structure of *S. cerevisiae* Spt4 complexed with the NGN domain of Spt5 and identified critical amino acids for this interaction, which are highly conserved in eukaryotes (Guo et al., 2008). We introduced corresponding amino acid substitutions into full-length *Drosophila* Spt5 (E265K) and Spt4 (S69F). Non-mutated and mutated versions were co-expressed as HA- (HA::Spt4, HA::Spt4^S69F^) and Myc-tagged proteins (Myc::Spt5, Myc::Spt5^E265K^) in *Drosophila* S2R cells. Co-immunoprecipitation demonstrated binding of wild-type Spt4 to Spt5, whereas the S69F substitution in Spt4 and the E265K substitutions in Spt5 disrupted this interaction (Figure 1A). For further analysis, we generated transgenic flies expressing HA::Spt4, HA::Spt4^S69F^, Myc::Spt5 and Myc::Spt5^E265K^ under UAS control. Transgenes were expressed with the neuroblast specific driver line worniu-Gal4 in combination with UAS-mCD8::GFP as a marker. In accordance with their function as transcriptional co-regulators, non-mutated HA::Spt4 and Myc::Spt5 co-localized in the nuclei of neuroblasts and their progenies (Figure 1B, top row). The S69F substitution in Spt4 resulted in re-distribution of the protein throughout the cell without affecting nuclear localization of Myc::Spt5 (Figure 1B, middle row). The uniform localization of Spt^S69F^ might be a consequence of passive diffusion. Alternatively, the N-terminal sequence (L-X_2_-L-X_3_-L-X_3_-L) of Spt4 closely conforms to the consensus nuclear export signal sequence (L-X_2-3_-L-X_2-3_-L-X-L).

The E265K substitution in Spt5 had no effect on its own nuclear localization. HA::Spt4 localization was only partially altered, probably because endogenous Spt5 captured HA::Spt4 at least to some degree in the nucleus (Figure 1B, bottom row). To verify that the S69F and the E265K substitutions disrupt association of Spt4 with Spt5 in flies, we performed proximity ligation assays (PLA). Nuclear PLA signals were seen upon co-expression of non-mutated Spt4 and Spt5 (Figure 1C), whereas in case of co-expression of HA::Spt4^S69F^ with Myc::Spt5 or HA::Spt4 with Myc::Spt5^E265K^ no or very few background signals were evident (Figure 1C). These results allowed us to conclude that Spt4-Spt5 complex formation takes place in the nucleus. This interaction is necessary to retain Spt4 in the nucleus, whereas Spt5 localization is independent of Spt4.

### Generation of Spt 4 and Spt5 knock-out flies

Previously we have shown that cell-type specific knock-down of Spt5 by RNAi delays tumor growth by reducing neuroblast proliferation (Hofstetter et al., 2024). However, this analysis was restricted to Spt5 and some phenotypes might not be expressed because of incomplete gene silencing. Therefore, we generated Spt4 and Spt5 deletion alleles (*Spt4* ^*Δ*^ and *Spt5* ^*Δ*^) by CRISPR mediated HDR. The *Spt4* ^*Δ*^ deletion encompasses nearly the complete open reading frame, whereas *Spt5* ^*Δ*^ removes part of the NGN domain, KOW domains 1-5 and the CTR (Supplementary Figure S1). As mentioned, the NGN domain and KOW domains 1, 4 and 5 are essential for the *in vivo* function of Spt5. *Spt4* ^*Δ*^ and *Spt5* ^*Δ*^ cause homozygous lethality at late embryonic/early first instar larval stages.

### *Spt4* ^*Δ*^ and *Spt5* ^*Δ*^ impair mushroom body neuroblast proliferation

**FIGURE 2:**
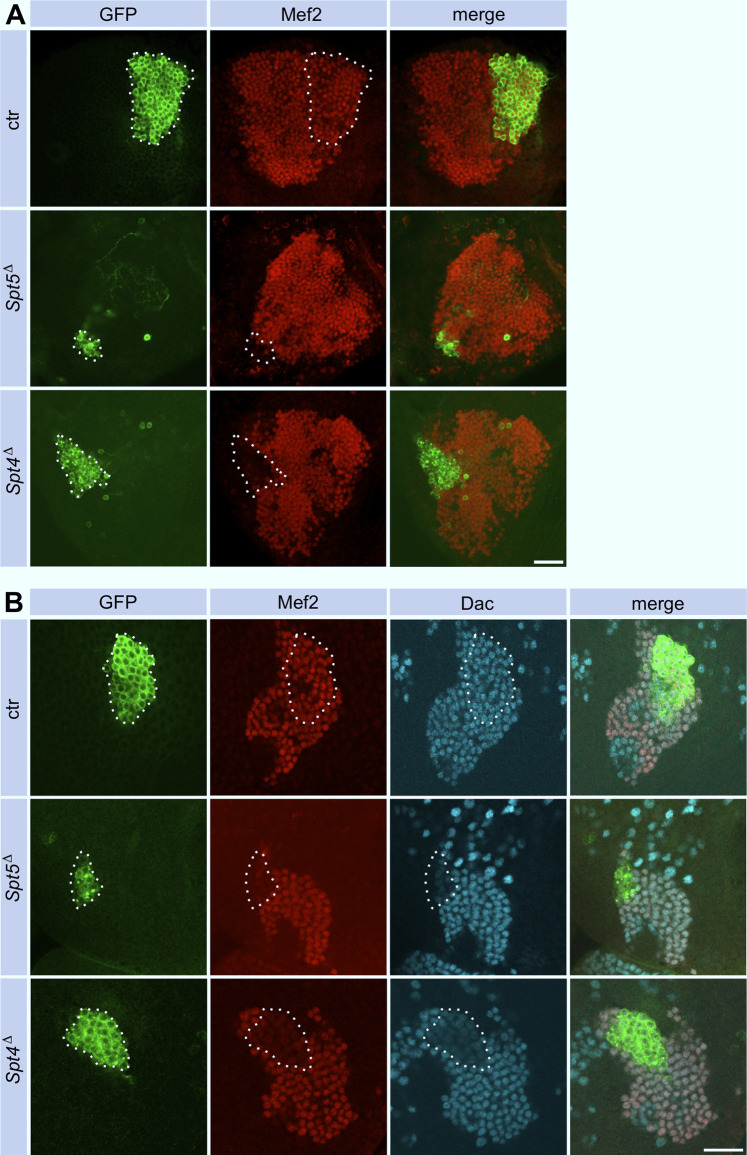
Spt5 and Spt4 deletion reduce Kenyon cell number. Control, *Spt5* ^*Δ*^ and *Spt4* ^*Δ*^ MBNB clones were induced in first instar larvae and analyzed in the adult **(A)** or in third instar larvae **(B)**. Clonal cells (encircled) were detected with an anti-GFP antibody (green), co-staining with antibodies against Myocyte enhancing factor 2 (Mef2, red) and Dachshund (Dac, cyan) labeled KC nuclei. For quantitative analysis see Supplementary Figure S2 (cell number), Supplementary Figure S4A (Dac) and Supplementary Figure S4B (Mef2). Scale bar: 20 μm.

As a model system to study the phenotypic consequences of Spt4 and Spt5 deletion, we used the bilateral arranged mushroom bodies in the central brain. The sequential generation of γ-, α´/β′- and α/β-neurons (Kenyon cells, KCs) from four equipotent mushroom body neuroblasts (MBNB) and their defined axonal projection patterns into a system of lobes allowed us to determine effects of *Spt4* ^*Δ*^ and *Spt5* ^*Δ*^ both on MBNB proliferation and differentiation of neurons using the clonal MARCM system to label individual MBNBs and their progenies (Lee et al., 1999). Induction of single MBNB clones in first instar larvae from control animals resulted in labeling of large KC clusters in adult brains (Figure 2A, for quantification see Supplementary Figure S2). In comparison, *Spt5* ^*Δ*^ mutant clones comprised only very few KCs (Figure 2A; Supplementary Figure S2). A less pronounced decrease in KC number was observed for *Spt4* ^*Δ*^ MBNB clones (Figure 2A; Supplementary Figure S2). Analysis of MBNB clones in third instar larval brains provided the same result with the strongest reduction in clone size seen for *Spt5* ^*Δ*^ (Figure 2B; Supplementary Figure S2). As MBNBs proliferate into mid-pupal stage, there is a corresponding increase in KC number in control clones from third instar larvae to the adult. Strikingly, *Spt4* ^*Δ*^ and *Spt5* ^*Δ*^ mutant clones not only contained fewer KCs, but also did not show the increase in KC number between larval and adult stages (Supplementary Figure S2). Both phenotypes indicated impaired and premature stop of cell proliferation of *Spt4* ^*Δ*^ and *Spt5* ^*Δ*^ mutant MBNB. In addition, survival of generated KCs could be affected.

**FIGURE 3:**
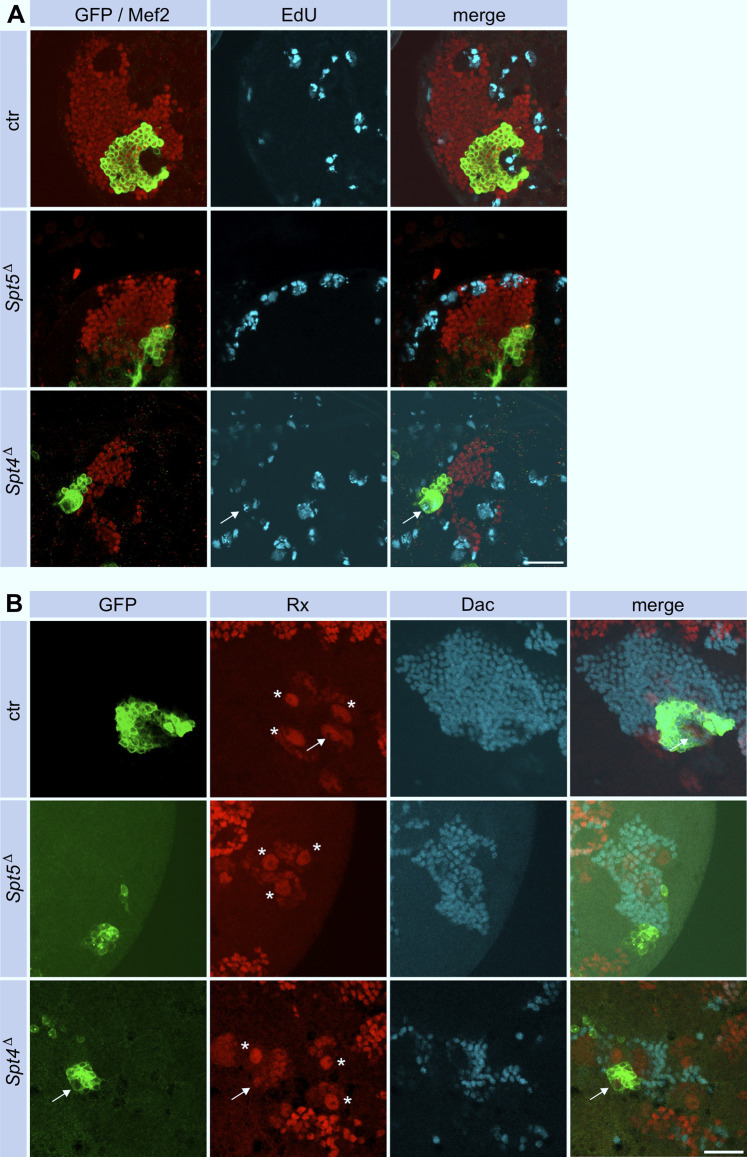
Spt5 and Spt4 deletion impair MBNB proliferation. **(A)** Control, *Spt5* ^*Δ*^ and *Spt4* ^*Δ*^ MBNB clones were induced in first instar larvae. At third larval instar, brains were dissected and replicating DNA was labeled with EdU for 90 min (cyan). Antibody stainings labeled clonal cells (GFP, green) and KC nuclei (Mef2, red). Scale bar: 20 μm. **(B)** In third instar larval brains from control animals, Rx staining labeled the four large MBNBs (stars and arrow) and associated smaller GMCs within the KC cell body layer (Mef2, red). A single MBNB (arrow) localizes within clonal cells (GFP, green). No Rx signal associated with clonal cells is seen for *Spt5* ^*Δ*^, faint Rx expression is evident in a smaller *Spt4* mutant MBNB (arrow). Dac was used as a general marker for KC nuclei. Scale bar: 20 μm.

To investigate these phenotypes in more detail, we induced *Spt4* ^*Δ*^ or *Spt5* ^*Δ*^ mutant MBNB clones in first instar larvae and analyzed brains from third instar larvae, a developmental stage when MBNB are still proliferating. Staining for the pro-apoptotic protein Dcp-1 provided no evidence for enhanced neuronal cell death upon Spt4 or Spt5 deletion (Supplementary Figure S3). Proliferation was assayed by pulse labeling of S-phase cells with the base analogue EdU. In control animals, EdU signals were seen throughout the brain, corresponding to continuously dividing neuroblasts and derived ganglion mother cells (GMCs), which divide only once to generate a pair of neurons. This also includes the four MBNB (and associated GMCs), one of which generates the GFP-labeled cell clone (Figure 3A). Within *Spt5* ^*Δ*^ mutant cell clones (Figure 3A), no EdU signals could be detected (0 out of 9 brains analyzed). In case of *Spt4* ^*Δ*^ mutant cell clones (Figure 3A), EdU incorporation was evident in most cases (7 out of 11 brains analyzed) in a single larger cell, which we consider as the neuroblast, and in some surrounding smaller cells corresponding to GMCs. These results indicated that between the time point of clone induction in first instar larvae and analysis in third instar larvae, MBNBs and/or GMCs stopped proliferation in case of deletion of Spt5, whereas removal of Spt4 function only impaired mitotic activity based on the smaller overall clone size. To directly monitor the presence of MBNBs and GMCs in the absence of Spt5 or Spt4 function, we performed stainings against Roughex (Rx), a transcription factor expressed among others in the nuclei of MBNBs and derived GMCs (Kraft et al., 2016). Four large Rx-positive signals representing the 4 MBNB nuclei surrounded by smaller labeled GMCs nuclei are seen in control brains with one Rx cluster localized in the GFP-marked clone (Figure 3B). In contrast, in 10 out of 12 brains analyzed, no Rx signal was associated with GFP labeled *Spt5* ^*Δ*^ cell clones (Figure 3B), only in two cases, we observed weak Rx expression in a small GFP-positive cell. In 7 out of 8 *Spt4* ^*Δ*^ mutant cell clones, Rx signals were evident. However, the single larger Rx signal belonging to the MBNB within the clone has a much smaller diameter than in the remaining 3 MBNBs outside of the clone (Figure 3B). Since decrease in neuroblast size correlates with the end of neurogenesis, this indicated an impairment of *Spt4* ^*Δ*^ mutant MBNBs to maintain proliferation.

In summary, Spt5 and, to a lesser extent, Spt4 are required for continuous cell division of MBNB. The results are consistent with our previous findings showing a negative effect of Spt5 knock-down on proliferation in a brain tumor model (Hofstetter et al., 2024).

### *Spt4* ^*Δ*^ and *Spt5* ^*Δ*^ show defects in γ-neuron remodeling

**FIGURE 4:**
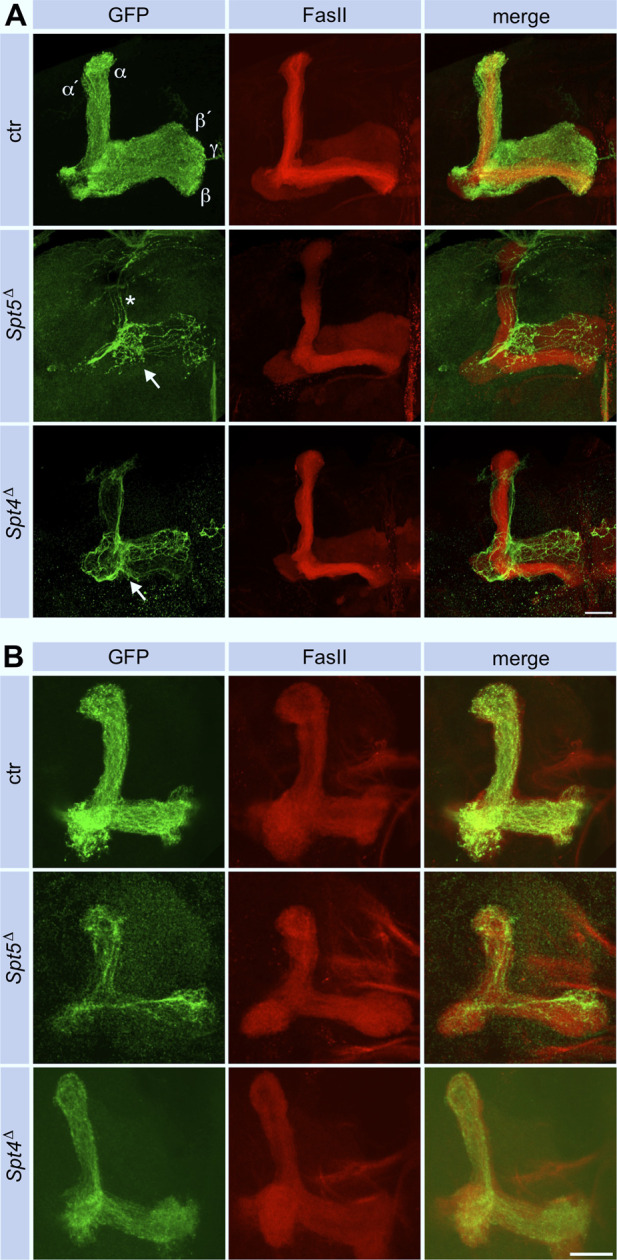
Spt5 and Spt4 are required for neuronal remodeling. Control, *Spt5* ^*Δ*^ and *Spt4* ^*Δ*^ MBNB clones were induced in first instar larvae and analyzed in adult **(A)** or in third instar larval brains **(B)**. Fasciclin II (FasII, red) labels the mushroom body lobe system (α/β, α´/β′and γ), with prominent staining of the adult α/β-lobes. GFP (green) labels the axons from clonal KCs, which project in all lobes in control animals, reflecting the ability of a single MBNB to sequentially generate all KC subtypes. In case of *Spt5* ^*Δ*^ and *Spt4* ^*Δ*^, most axons stopped before entering the lobe system (arrows), only few reached the γ-lobe. In *Spt5* ^*Δ*^, some remnants of dorsal projections are visible (star), in case of *Spt4* ^*Δ*^, these projections follow the α´/β′-lobe. Scale bar: 20 μm. **(B)** Deletion of either Spt4 or Spt5 had no effect on projection of KC axons into the larval lobe system stained for FasII. Projections are sparser because of the proliferation defect of the mutant MBNBs. Scale bar: 20 μm.

Since MBNB clones were induced at first instar larvae, GFP labeled cells in adult control brains belong to the sequentially generated γ-, α´/β′- and α/β-KCs with their axonal projections into the corresponding lobes (Figure 4A). The finding that *Spt5* ^*Δ*^ mutant MBNB stopped proliferation already in third instar larvae predicted that the few neurons generated by such a neuroblast should mainly belong to the γ-KC class forming the medial γ-lobe. These neurons are unique in terms of their differentiation as they remodel their axonal projections during metamorphosis. Larval γ-neuron axons project through the peduncle and then branch into a medial and dorsal lobe. With beginning of metamorphosis, the axonal branches are pruned, and single projections regrow to form the medial γ-lobe of the adult mushroom body. In case of Spt5 deletion, adult axonal projections follow the peduncle but then most of them remain confined there and only some project into the γ-lobe (Figure 4A). Very few thin dorsal projections were also visible (Figure 4A, star in middle row); these might be the remains of larval γ -KC axons which failed pruning or rare α´/β′-KCs still produced by the mutant MBNB.

We could not distinguish between these possibilities because usage of two γ-KC specific driver lines (*H24-Gal4* and *GMR71G10-Gal4*) instead of the pan-KC driver line *ey* ^*OK107*^ *-Gal4* labeled no cells in Spt5 MBNB clones (data not shown). This indicated a general requirement of Spt5 for normal γ-neuron differentiation. Indeed, Spt5 deletion caused downregulation of two transcription factors involved in KC differentiation, Dachshund (Dac) (Martini et al., 2000) and Myocyte enhancing factor 2 (Mef2) (Crittenden et al., 2018) in larval (Figure 2B, for quantification see Supplementary Figures S4A, SB) and adult brains (Figure 2A). To confirm or exclude a general differentiation defect as the cause for proper axon outgrowth of γ-neurons in adult brains, we performed two experiments. First, when *Spt5* ^*Δ*^ mutant clones were induced in first instar larvae and analyzed in third instar larvae, the few generated γ-KCs had a normal projection pattern with a vertical and a medial branch (Figure 4B), which argues against a general function of Spt5 in γ-KC specification and in initial outgrowth of their axons. On the other hand, conditional knock-down of Spt5 with a *UAS-shRNA-Spt5* construct expressed in γ-KCs only in the time frame of neuronal remodeling using the *H24-Gal4* or *GMR71G10-Gal4* driver lines in combination with a temperature sensitive *Gal80* transcriptional repressor line (shifted to the restrictive temperature from late third instar larval stage onwards) resulted in no reproducible axon outgrowth phenotype (data not shown). We do not know whether this failure is due to inefficient Spt5 knock-down, perdurance of existing Spt5 protein or indeed reflects an earlier function of Spt5 in γ-neuron differentiation required for subsequent remodeling.

Performing the same experiments with *Spt4* ^*Δ*^ re-capitulated the phenotypes of *Spt5* ^*Δ*^, but some defects were not as pronounced. Dac and Mef2 expression were decreased to a similar degree in clonal cells (Figure 2B; Supplementary Figures S4A, SB). As shown, Spt4 deficient MBNBs generated less KCs (Supplementary Figure S2). Most of them project into the γ-lobe and only few into the α´/β′-lobe (Figure 4A), again reflecting impaired proliferation and precocious termination of neurogenesis before α/β-KCs are born. Larval γ-KC axons branched normally into the vertical and medial lobe (Figure 4B), but after pruning, minor projection defects into the adult γ-lobe were observed (Figure 4A).

### *Spt4* ^*Δ*^ and *Spt5* ^*Δ*^ affect expression of genes required in neuronal remodeling

**FIGURE 5:**
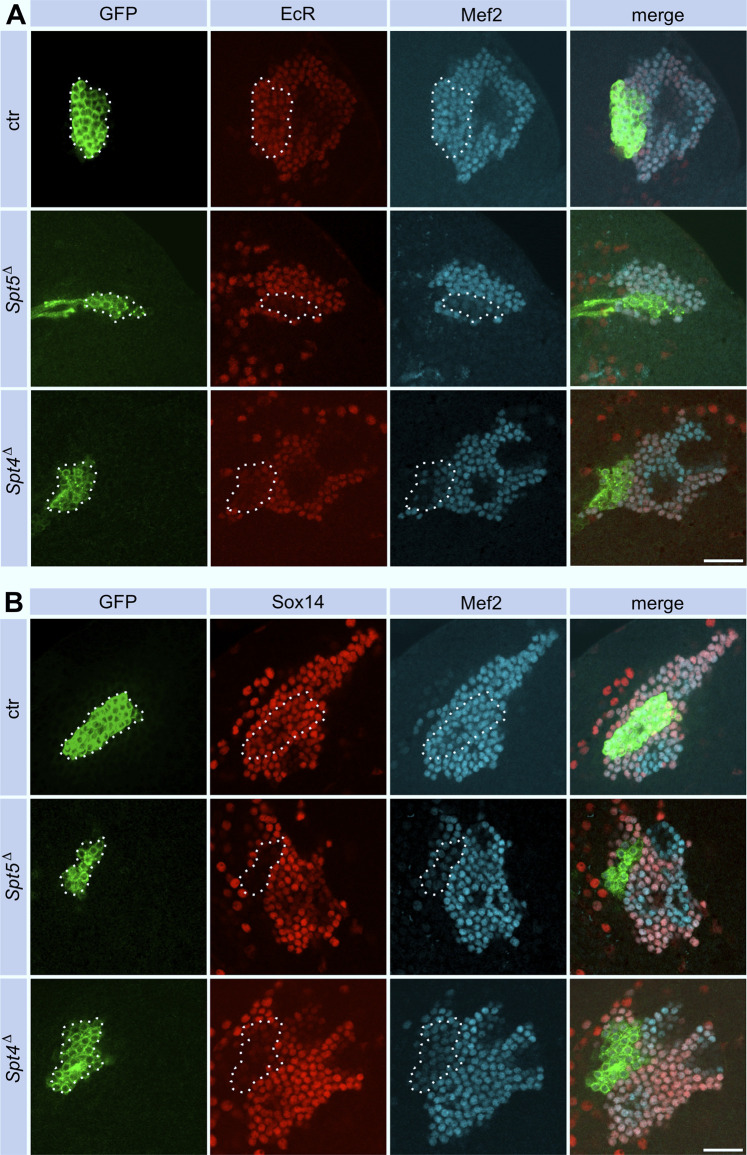
Spt5 and Spt4 deletion interferes with Ecdysone signaling. Control, *Spt5* ^*Δ*^ and *Spt4* ^*Δ*^ MBNB clones were induced in first instar larvae and brains were analyzed at third larval instar for expression of EcR B1 (A, red) and Sox14 (B, red). Co-staining for GFP (green) labeled clonal cells (encircled), Mef2 **(A)** and Dac **(B)** were used as nuclear KC markers (cyan). For quantitative analysis see Supplementary Figure S4C (EcR) and Supplementary Figure S4D (Sox14). Scale bar: 20 μm.

Pruning and regrowth of γ-KC axons correlate with significant changes in the transcriptional program (Alyagor et al., 2018). Given the role of Spt4 and Spt5 as transcriptional co-regulators and the central role of Ecdysone-triggered transcriptional changes during remodeling (Yaniv and Schuldiner, 2016; Furusawa and Emoto, 2021; Truman and Riddiford, 2023), we evaluated changes in the expression level of the relevant Ecdysone receptor isoform B1 (EcR B1) and one of its targets, the transcription factor Sox14. Both proteins were reduced in Spt5 and Spt4 depleted KCs (Figures 5A, B, for quantification see Supplementary Figures S4C, SD). These observations correlated with previous findings that knock-down of EcR and Sox14 resulted in remodeling defects (Alyagor et al., 2018). We also noticed that EcR B1 was more evenly distributed in mutant cells, in contrast to its co-localization with nuclear Mef2 in non-mutant cells. This might indicate a failure in ligand mediated EcR B1 activation and its subsequent translocation into the nucleus to drive transcription of target genes.

**FIGURE 6:**
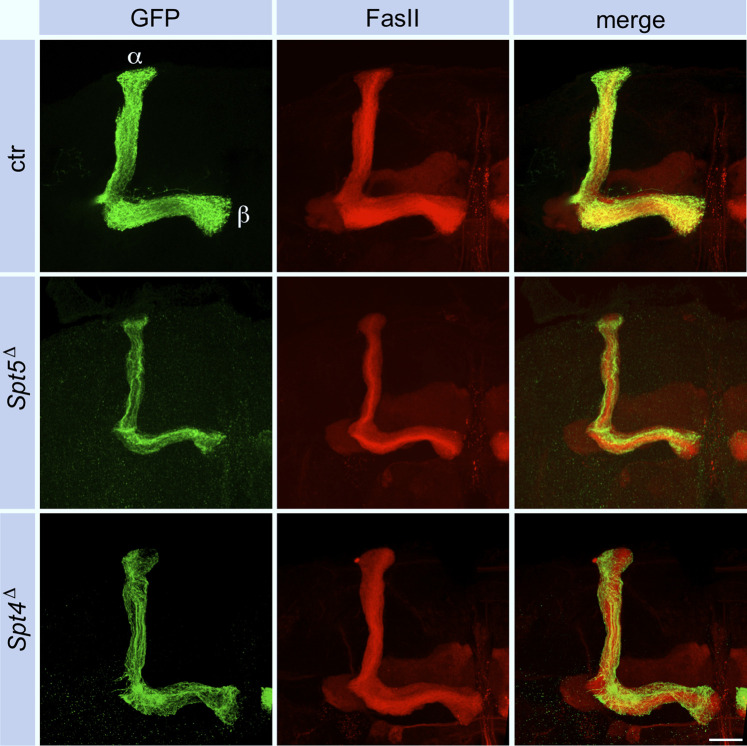
Spt5 and Spt4 deletion has no influence on axonal projections of α/β-KCs. Control, *Spt5* ^*Δ*^ and *Spt4* ^*Δ*^ MBNB clones were induced in late third instar larvae and the axonal projection pattern of clonal KCs (GFP, green) into the adult lobe system labeled for FasII (red) was analyzed. Because of the proliferation defect of the mutant MBNBs, the projections into the α/β-lobes were sparser but otherwise unaffected. Scale bar: 20 μm.

Since Spt4 and Spt5 mutant MBNBs precociously terminate proliferation, the question remained, whether Spt4 and Spt5 are required for specification and normal axonal differentiation of later born KCs. Therefore, Spt4 and Spt5 depleted MBNB clones were induced in late third instar larvae, the time from which the last born α/β-KCs are generated. In both mutant cases, α/β-KCs showed a wild-type axonal projection pattern (Figure 6), but projections were sparser reflecting the reduced number of generated neurons. These results again confirmed a function of Spt4 and Spt5 in cell proliferation and indicated that both proteins are not required for KC subtype specification. Further support for the latter notion came from the analysis of transcription factor Chronologically inappropriate morphogenesis (Chinmo) and its upstream posttranscriptional regulator IGF-II mRNA-binding protein (Imp). The progressive decline of Chinmo and Imp expression triggers the sequential generation and specification of γ-, α´/β′- and α/β-KCs (Zhu et al., 2006; Liu et al., 2015). In addition, Chinmo acts as one of the upstream regulators of EcR B1 expression (Marchetti and Tavosanis, 2017). Looking in third instar larval brains, Spt4 and Spt5 deletion had no effect on Imp (Supplementary Figures S3A, S4E for quantification) and Chinmo (Supplementary Figures S3B, S4F for quantification) expression levels. Although we did not analyze the decline in expression of Chinmo and Imp at later developmental stages, these findings support the hypothesis that Spt5 and Spt4 are not required for Imp-Chinmo dependent specification of KC subtypes. Furthermore, the observed decrease in EcR B1 levels upon Spt4 and Spt5 deletion (Figure 5A) is not caused by loss of Chinmo expression.

Taken together, Spt5 and Spt4 are required for proper expression of proteins involved in remodeling of γ-KC axons during metamorphosis rather than being generally required in axonal pathfinding.

## Discussion

As mentioned in the introduction, only in few cases, Spt4 or Spt5 were associated with specific developmental processes or diseases. The aim of this study was to perform a comparative phenotypic analysis of Spt4 and Spt5 knock-out alleles using the *Drosophila* mushroom body development as a model to address two main questions. First, in this specific cellular context, do Spt4 and Spt5 always act together, or do they also fulfill independent functions? Second, does deletion of either gene globally disturb mushroom body development or only specific aspects? In summary, all Spt5 mutant phenotypes were also observed in case of Spt4 deletion, although they were generally less pronounced. This includes the precocious termination of MBNB proliferation, the distinct effects on gene expression and the neuronal remodeling defect.

One explanation for the slightly different phenotypes could be found in the molecular properties of the two proteins. The large Spt5 protein plays a central role in transcription by making multiple contacts with DNA, RNA, Pol II and regulatory proteins, thereby controlling pausing, elongation and termination. Together with Spt5 and Pol II, the small Spt4 protein forms the DNA exit tunnel. In contrast to Spt5, Spt4 is not required for Pol II stability. Spt4 also facilitates transcription through nucleosomal barriers (Decker, 2021; Song and Chen, 2022). Although both proteins are essential for viability, their contribution to efficient and regulated transcription might be different. For example, the functional relevance of Spt4 seems to depend on the genomic context as it selectively regulates expression of genes with expanded hexanucleotide repeats (Cheng et al., 2015; Kramer et al., 2016; Furuta et al., 2019; Deng et al., 2022).

Which role could Spt5 and Spt4 play in maintaining continuous MBNB proliferation from embryogenesis until end of neurogenesis at mid-pupal stages? Spt5 depletion commenced at the onset of larval development resulted in loss of MBNB in third instar larvae. Proliferating Spt4 mutant MBNBs were still present at this developmental stage, but their cell size was much smaller compared to wildtype. Previous studies demonstrated a positive correlation between cell size and neuroblast proliferation. Regrowth after each cell division maintains the proliferation potential until the end of neurogenesis, where neuroblasts reduce size followed by terminal differentiation or, in case of MBNBs, cell death (Maurange et al., 2008; Siegrist et al., 2010; Homem et al., 2014). Cell growth requires ribosome biogenesis and protein synthesis, and one major regulator for expression of the corresponding genes is the transcriptions factor Myc (Grewal et al., 2005). Mutations in *Drosophila* Myc (dMyc) show profound growth defects at the organismal and cellular level, including neuroblasts (Song and Lu, 2011; Gallant, 2013; Rust et al., 2018). Compared to other brain neuroblasts, MBNBs are larger and have elevated dMyc levels, which contribute to their extended proliferation period (Samuels et al., 2020). The RNA binding protein Imp controls dMyc levels by stabilizing *dMyc* mRNA. The progressive decline of Imp levels not only regulate neuronal temporal fate but also decommissioning of neuroblasts (Liu et al., 2015; Yang et al., 2017; Samuels et al., 2020). Since Spt5 or Spt4 deletion did not influence Imp protein levels at least in third instar brains (Supplementary Figure S3A), we consider a more direct involvement of both proteins in Myc-mediated transcription as a likely explanation. Recent studies in human cell lines showed that Myc recruits Spt5 and hands it over to RNA Polymerase II to promote processive transcription elongation (Baluapuri et al., 2019). In cell culture experiment, we confirmed interaction of *Drosophila* Spt5 and dMyc (T.R., data not shown). Thus, in the absence of Spt5/Spt4, growth signals mediated by dMyc might not be efficiently translated into productive transcription to maintain proper neuroblast size.

Finally, our analysis implicated a function of Spt4 and Spt5 in neuronal remodeling of γ-KC. However, the failure to reproduce the phenotype caused by Spt5 and Spt4 deletion alleles by conditional Spt5 knock-down specifically in the time frame of remodeling leaves open the question, whether knock-down was inefficient or whether Spt5 has an impact on proper γ-KC differentiation before remodeling is initiated. There are arguments for both scenarios. The initial elaboration of γ-KC axons was normal but then they failed after pruning to regrow into the adult γ-lobe. Also, loss of Spt4 or Spt5 function has no influence on the establishment and maintenance of the axonal projection patterns of α´/β′-and α/β-KCs. This would argue for proper initial differentiation of all KC and a requirement of Spt4 and Spt5 at the time point of axonal re-organization of γ-KC. On the other hand, expression of two transcription factors required for KC differentiation, Dachshund (Dac) (Martini et al., 2000) and Myocyte enhancing factor 2 (Mef2) (Crittenden et al., 2018), are prominently reduced already in L3 larvae and this might in turn influence expression of genes required for neuronal remodeling at the onset of pupal stage.

Looking for a molecular explanation we found out that expression of two components of the Ecdysone signaling pathway required for neuronal remodeling, the receptor EcR B1 and its transcriptional target Sox 14, were reduced in their expression. Spt5 and Spt4 could either directly regulate EcR B1 gene expression or indirectly by influencing transcription of upstream regulators of EcR B1 expression, also before remodeling in initiated. EcR expression is under control of several systems: TGF-β signaling, the orphan nuclear receptor FTZ-F1, the cohesion complex, micro-RNAs (Yaniv and Schuldiner, 2016; Furusawa and Emoto, 2021) and the transcription factor Chinmo (Marchetti and Tavosanis, 2017). So far, we could only exclude a major influence of Spt4 and Spt5 on expression of Chinmo and its upstream regulator Imp. A related question remains open for Sox14. Reduced Sox 14 expression could be a consequence of decreased EcR B1 signaling. Alternatively, a direct influence of Spt4 and Spt5 on Sox 14 transcription is possible.

Even though a systematic analysis of the transcriptional targets of Spt4 and Spt5 in the context of mushroom body neurogenesis is still pending, our results support the idea both proteins work together to control differential gene expression and thereby elicit cell-type specific responses.

## Data Availability

The raw data supporting the conclusions of this article will be made available by the authors, without undue reservation.
