# Supplementary figures and images for: The transcription elongation factors Spt4 and Spt5 control neural progenitor proliferation and are implicated in neuronal remodeling during Drosophila mushroom body development
Source: Front Cell Dev Biol. 2024 Oct 9;12:1434168. doi: 10.3389/fcell.2024.1434168 (PMC11496258; doi:10.3389/fcell.2024.1434168)

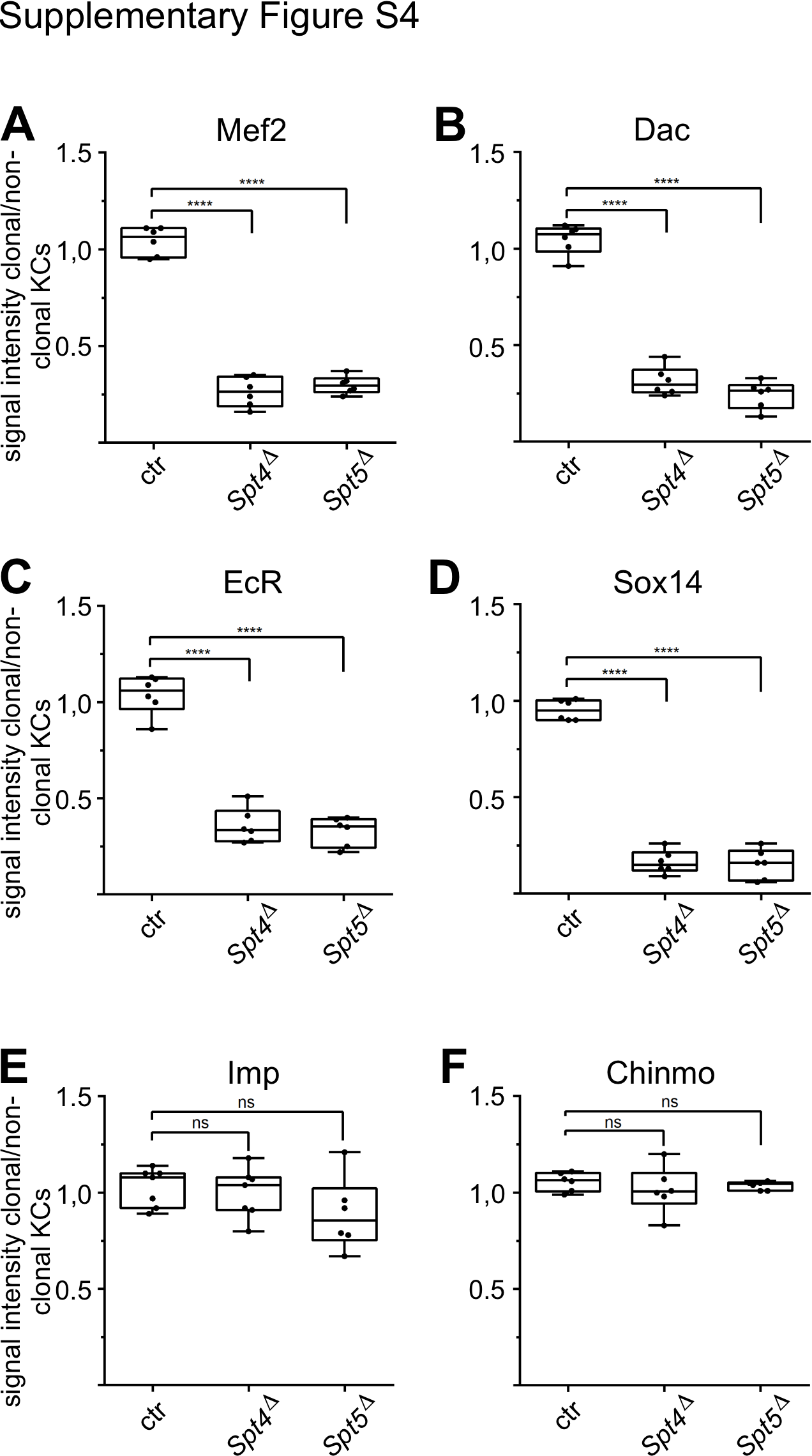

Supplement: Supplementary file 2 [file Image4.tif]

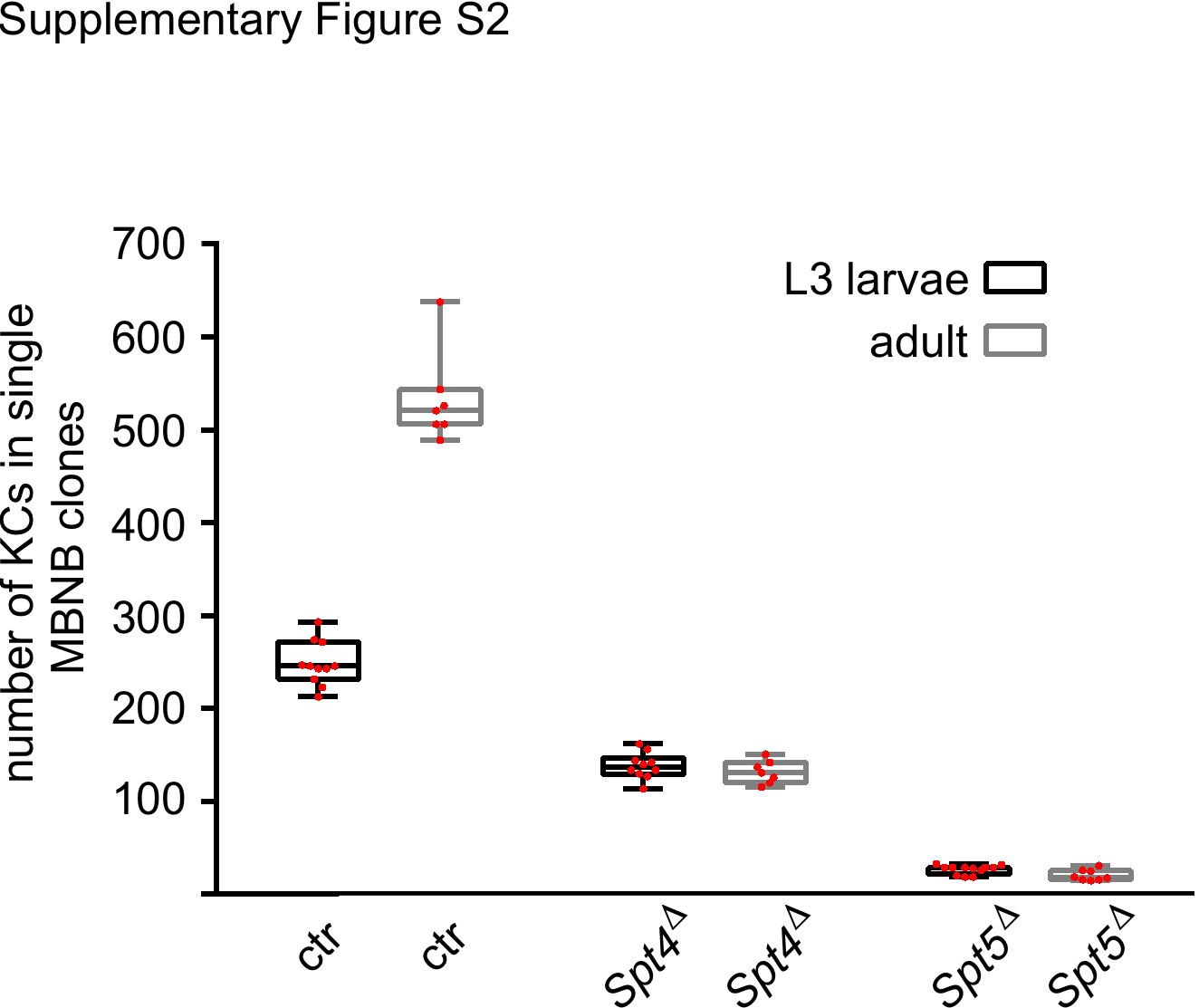

Supplement: Supplementary file 3 [file Image2.tif]

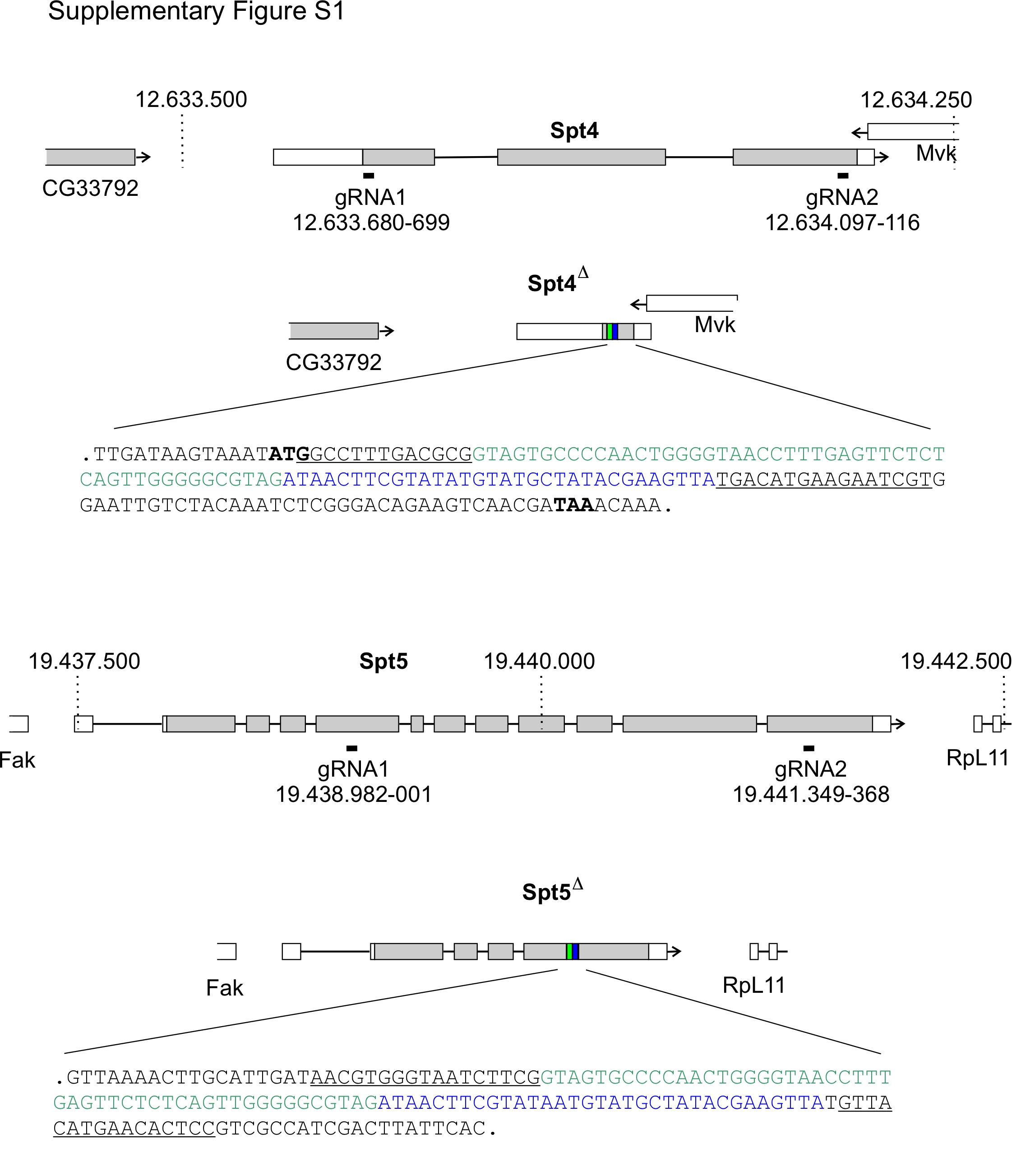

Supplement: Supplementary file 5 [file Image1.jpg]
