# Supplementary material for: The transcription elongation factors Spt4 and Spt5 control neural progenitor proliferation and are implicated in neuronal remodeling during Drosophila mushroom body development
Source: Front Cell Dev Biol. 2024 Oct 9;12:1434168. doi: 10.3389/fcell.2024.1434168 (PMC11496258; doi:10.3389/fcell.2024.1434168)
Supplement: Supplementary file 6 [file DataSheet1.docx]

**Supplementary Material**

**Genotypes of flies in Figure 1C**

*w; worniu-Gal4, UAS-mCD8::GFP/+; UAS-HA::Spt4/UAS-Myc::Spt5*

*w; worniu-Gal4, UAS-mCD8::GFP/+; UAS-HA::Spt4^S69F^/UAS-Myc::Spt5*

*w; worniu-Gal4, UAS-mCD8::GFP/+; UAS-HA::Spt4/UAS-Myc::Spt5^E265K^*

**Genotypes of flies in Figures 2, 3, 4, 5, 6, Supplementary Figures S2, S3, S4, S5**

*Ctr: y,w,hsP-FLP/+; FRT(G13)42B, UAS-mCD8::GFP/FRT(G13)42B, tubP-Gal80; ey^OK107^-Gal4/+*

*Spt5^Δ^: y,w,hsP-FLP,UAS-mCD8::GFP/+; FRT(G13)42B,Spt5^Δ^/FRT(G13)42B,tubP-Gal80; ey^OK107^-Gal4/+*

*Spt4^Δ^*: *y,w,hsP-FLP,UAS-mCD8::GFP/+; FRT(G13)42B,Spt4^Δ^/FRT(G13)42B,tubP-Gal80; ey^OK107^-Gal4/+*

**Supplementary Figure legends**

**Supplementary Figure S1: Generation of *Spt4^Δ^* and *Spt5^Δ^* alleles by CRISPR mediated HDR**.

Genomic organization of the Spt4 and Spt5 gene loci on the 2^nd^ chromosome. Genomic numbering according to FlyBase (version R6.56). Exons are represented as boxes, grey highlights coding sequences. The genomic positions of the gRNAs used for integration of the donor construct are indicated. Flanking loxP sequences allowed removal of the dsRed marker by Cre recombinase resulting in the final *Spt4^Δ^* and *Spt5^Δ^* alleles. Sequences from PCR amplified genomic DNA from these flies are shown below. The remaining single loxP site (blue), the attP integration site of the donor vector (green), the remaining sequences recognized by the gRNAs (underlined) and start respectively stop codon (bold) of Spt4 are highlighted.

**Supplementary Figure S2: Reduction of KC number in *Spt4^Δ^* and *Spt5^Δ^* mutant MBNB clones.**

Counting of KC number (GFP+ labeled cells in single MBNB clones) in late 3^rd^ instar larval brains (L3) or in adults. L3 control: 248 ± 23 (SD), n=10**;** L3 *Spt4^Δ^*: 138 ± 14 (SD), n=10; L3 *Spt5^Δ^*: 26 ± 4 (SD), n=12; adult control: 533 ± 49 (SD), n=7; adult *Spt4^Δ^*: 132 ± 12 (SD), n=7; and adult *Spt5^Δ^*: 21 ± 6 (SD), n=8.

**Supplementary Figure S3: Loss of Spt4 and Spt5 does not enhance apoptosis.**

KCs derived from *Spt5^Δ^* and *Spt4^Δ^* MBNBs (GFP, green) were assayed for enhanced apoptosis using an antibody against the cleaved (active) effector caspase Dcp-1 (red). Dac served as a marker for KC nuclei (cyan). No Dcp-1 signals were visible in clonal cells. Scale bar: 20μm.

**Supplementary Figure S4: Effect of *Spt4^Δ^* and *Spt5^Δ^* on protein expression levels in KCs.**

Protein expression levels were determined in GFP labeled KCs in control, *Spt4^Δ^* and *Spt5^Δ^* MBNB clones and set in relation to the expression level in non-clonal KCs located in close proximity. At least 6 brains were analyzed for each genotype and staining for Mef2 (A, related to Figure 2B), Dac (B, related to Figure 2B), EcR (C, related to Figure 5A), Sox14 (D, related to Figure 5B), Imp (E, related to Supplementary Figure S5) and Chinmo (F, related to Supplementary Figure S5).

**Supplementary Figure S5: Loss of Spt4 and Spt5 does not affect the KC specification determinants Imp and Chinmo.**

3^rd^ instar larval brains with GFP-labeled KC clones (green) derived from control, *Spt5^Δ^* and *Spt4^Δ^* MBNBs induced in 1^st^ instar larvae were stained for Imp (A, red) or Chinmo (B, red). Dac (A) and Mef2 (B) were used as general nuclear KC markers (cyan). Imp and Chinmo expression were not changed in clonal KC cells (encircled) in comparison to surrounding KCs. For quantitative analysis see Supplementary Figure S4E (Imp) and S4F (Chinmo). Scale bar: 20μm.
